# Spatio-Temporal Effects of Multi-Dimensional Urbanization on Carbon Emission Efficiency: Analysis Based on Panel Data of 283 Cities in China

**DOI:** 10.3390/ijerph182312712

**Published:** 2021-12-02

**Authors:** Zhanhang Zhou, Linjian Cao, Kuokuo Zhao, Dongliang Li, Ci Ding

**Affiliations:** 1 School of Economics and Management, Tianjin Chengjian University, Tianjin 300384, China; zhouzhanh@163.com (Z.Z.); ldlessay@163.com (D.L.); dingci98@126.com (C.D.); 2 Research Center for Urbanization and New Rural Construction, Tianjin Chengjian University, Tianjin 300384, China; 3 School of Management, Guangzhou University, Guangzhou 510006, China; zhaokuok@126.com

**Keywords:** urbanization, carbon emission efficiency, US-SBM, Spatial Markov chains, GTWR

## Abstract

Under the influence of complex urbanization, improving the carbon emission efficiency (CEE) plays an important role in the construction of low-carbon cities in China. Based on the panel data of 283 prefectural-level cities in China from 2005 to 2017, this study evaluated the CEE by the US-SBM model, and explored the spatial agglomeration evolution characteristics of CEE from static and dynamic perspectives by integrating ESDA and Spatial Markov Chains. Then, the spatial heterogeneity of the impacts of multi-dimensional urbanization on CEE were analyzed by using the Geographically and Temporally Weighted Regression (GTWR). The results show that: (1) with the evolution of time, the CEE has a trend of gradual improvement, but the average is 0.4693; (2) from the perspective of spatial static agglomeration, the “hot spots” of CEE mainly concentrated in Shandong Peninsula, Pearl River Delta, and Chengdu-Chongqing urban agglomeration; The dynamic evolution of CEE gradually forms the phenomenon of “club convergence”; (3) urbanization of different dimensions shows spatial heterogeneity to CEE. The impact of economic urbanization in northern cities on CEE shows an inverted “U” shape, and the negative impact of spatial urbanization on CEE appears in the northwest and resource-based cities around Bohai Sea. Population and social urbanization have a positive promoting effect on CEE after 2010. These findings may help China to improve the level of CEE at the city level and provide a reference for low-carbon decision-making.

## 1. Introduction

In recent years, global warming, glacier retreat, and other climate change issues have aroused widespread concern. In the fourth Global Climate Assessment, the Intergovernmental Panel on Climate Change (IPCC) pointed out that human activities and massive emissions of greenhouse gases are the main causes of global climate change [1]. Existing studies have proved that the greenhouse effect is mainly caused by excessive carbon emissions, of which cities are one of the major contributors [2]. Urban areas, while generating nearly 2/3 of the world’s wealth, also emit 3/4 of the world’s total emissions and produce 4/5 of the world’s environmental pollution [3]. In the face of climate change, the sustainability of urban development is challenged. The United Nations 2030 Agenda for Sustainable Development shows that nearly 60 percent of the world’s population (about 5 billion people) will live in urban areas by 2030. In the coming decades, about 95% of urban expansion will take place in developing countries, where energy demand is high, and efficiency still has much potential to improve. Reducing carbon emission and improving carbon emission efficiency (CEE) are urgent goals for all mankind [4].

To achieve sustainable development, countries have proposed low-carbon action plans, such as the low-carbon action plan in the United Kingdom and the low-carbon society action plan in Japan [5]. As the world’s largest carbon emitter, China is trying to reduce its emissions, but its rapid urbanization has challenged its efforts to do so. China is also the most populous country, accounting for about 20% of the global population [6]. According to the current development trend, by 2050, China’s urban population will increase by 300–400 million, and the population urbanization rate will reach about 70% [7]. The rapid industrialization process will lead to a contradiction between urbanization and carbon emission reduction [8]. As a responsible country to cope with climate change, China has made a series of efforts. For example, in 2014, the Chinese government proposed to build a new type of urbanization characterized by industrial interaction, conservation and intensification, ecological livable, and harmonious development. Low-carbon city construction is an important connotation of urbanization development. Therefore, as the largest emitter, urbanization and carbon emission reduction will be two major features of China’s economic development in the future, and improving CEE will be an effective means of emission reduction at the current stage [9].

Urbanization is a complex economic phenomenon. It not only means the flow of rural population to cities, but also means the changes of population lifestyle, economic development mode, land use mode, and many other aspects [10]. In order to improve the CEE in the process of urbanization, it is necessary to clarify the CEE evolution trend and spatial dynamic characteristics, which will provide guidance for urban managers to formulate efficient and low-carbon policies. Although the relationship between urbanization and CEE has been confirmed in existing literature, the influence of different urbanization subsystems (population, economy, spatial and social urbanization) at the prefecture level in China on various mechanisms of CEE has not been fully discussed.

Therefore, this study aimed to achieve the following three main objectives: (1) measure the CEE of Chinese cities; (2) explore the change characteristics of the spatial pattern of CEE from static agglomeration and dynamic transfer; and (3) explain the impact of urbanization on carbon emission efficiency from time and space dimensions, and propose suggestions to improve the efficiency of carbon emission.

## 2. Literature Review

With the increasingly serious global warming, relieving the environmental pressure brought by rapid economic growth has become the focus around the world. Scientific measurement of CEE is the basis for formulating emission reduction targets and policies and promoting overall improvement of environmental quality [11]. There are “single factor” and “total factor” methods to calculate CEE. The single-factor method is mainly used for carbon productivity [12], carbon emissions per unit GDP [13,14], carbon index [15], and other indicators to measure CEE, with the advantages of simple calculation, easy operation, and understanding, but they also have obvious defects. For example, the energy intensity index only reflects the impact of energy on economic output, without taking capital, labor, and other factors into account [16].

Most existing studies suggest that capital, labor, GDP, and others should be considered comprehensively when constructing the carbon emission efficiency index. Hu and Wang applied the DEA method to the construction of the total factor energy efficiency index and calculated the energy saving and emission reduction capacity of China [17]. Later, researchers found that the traditional DEA model lacked the ability to calculate bad output, and the new DEA model was developed. For example, Anser et al. used mathematical knowledge to improve the ability of the DEA method to analyze environmental performance by comparing radial and non-radial technologies in environmental performance measurement [18]. Xie et al. adopted the DEA method to compare the carbon emission allocation amount and actual emissions in Chinese provinces, and concluded that the carbon emission utilization rate gradually increases from east to west regions [19].

Due to the differences in the urban development degree among different regions, CEE also presents the characteristics of spatial differentiation [8]. Some studies have analyzed spatial heterogeneity based on Theil index [5], coefficient of variation [20] and other exploratory spatial analysis methods, which are suitable for providing information of the whole region. However, these research methods rely on static processes to reflect regional phenomena, ignore the dynamic characteristics of regions, and fail to reflect internal dynamic information. In order to overcome this defect, some important studies used the Markov chain model to regard the spatial-temporal dynamic evolution of regional phenomena as a Markov process to achieve dynamic analysis [21,22]. For example, Losiri et al. simulated urban expansion and land use in Bangkok by using automata-Markov Chain (CA-MC) and multi-layer Perceptron-Markov Chain (MLP-MC) models [23]. Hamdy et al. explored the future urban expansion of Aswan region by combining Markov chain and Logistic regression, and argued that the risk of urban sprawl is increasing [24]. Wang et al. obtained the “club convergence” effect of CEE of Chinese cities through spatial Markov transfer probability matrix analysis [5]. Babbar used the Markov chain and InVEST model to evaluate carbon sequestration based on total carbon change under two different scenarios [25]. Ren predicted China’s energy consumption in 2030 by combining the GM (1,1) method with Markov chain [26].

**Figure 1:**
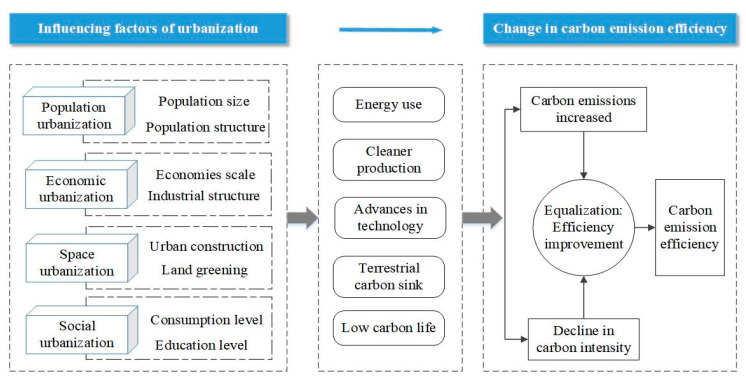
Influence mechanism of urbanization on carbon emission efficiency.

However, as one of the factors that has a significant impact on carbon emissions, CEE dynamic changes under the background of urbanization are rarely studied. Existing studies have focused more on the relationship between urbanization and carbon emissions [27]. Some studies argued that the expansion of the population will increase the demand for energy, thus increasing energy carbon emissions and reducing CEE [28]. Wang and Wang found that population aging can reduce carbon emissions and improve CEE in the long run [29]. Others believe that when per capita GDP is high enough, people will pursue more high-quality development, and production and life are gradually low carbon, which is conducive to the improvement of CEE [30]. Tao et al. and Xu et al. found that the improvement of land urbanization quality can promote the improvement of CEE [31,32]. In addition, some research believes that residents will have a lesser need for private transportation in rapidly urbanizing areas, thus reducing energy use and carbon emissions, leading to higher CEE [33]. Some studies have also discussed the impact of the COVID-19 pandemic on carbon emissions, as population consumption, travel, industrial production, and so on have been restricted during this period, especially in urban areas [34,35,36]. Capolongo et al. argued that with the COVID-19 epidemic, national containment measures have changed the lifestyles of people and communities and significantly reduced air pollution, and CEE also increased during this period [37]. In fact, there are many factors influencing the CEE, as shown in Figure 1.

In conclusion, a large number of studies have explored the influencing factors of CEE in China, providing suggestions for policy making. However, there are still limitations, highlighting the following research gaps: (1) the existing literature has mostly focused on single regions, such as provinces, and few have discussed the spatial agglomeration and spatial transfer of urban CEE in the whole country. In China, prefecture-level cities have always been regional political, economic, and cultural centers, accounting for a large proportion of the population and GDP [38]; (2) existing studies mainly focused on the calculation of the new-type urbanization index, and lack analysis of regional differences and spatial-temporal variation characteristics of the impact of each dimension of urbanization on carbon emissions; (3) in terms of analyzing the influencing factors of CEE, previous literature has mainly adopted traditional econometric methods [39,40], without considering the spatio-temporal heterogeneity. Chinese cities have significant differences in development, and the same influencing factors may have different impacts in different regions [41]. In addition, China’s “low-carbon city pilot” and other policies have made energy consumption and environmental pollution show a certain stage characteristic.

Therefore, this study takes 283 prefecture-level cities in China as the research object and studies the spatio-temporal heterogeneity of impacts of urbanization in different dimensions on CEE. To achieve these goals, we: (1) adopt the US-SBM model to calculate the CEE of Chinese cities from the perspective of total factors; (2) integrate ESDA and Spatial Markov chains to discuss the spatial agglomeration and type evolution characteristics of CEE from static and dynamic perspectives; and (3) analyze the relationship between urbanization and carbon emission efficiency in various dimensions by using spatiotemporal geographically weighted regression (GTWR) model, and regional differences with time and local geographical location changes are also discussed.

## 3. Study Area

**Figure 2:**
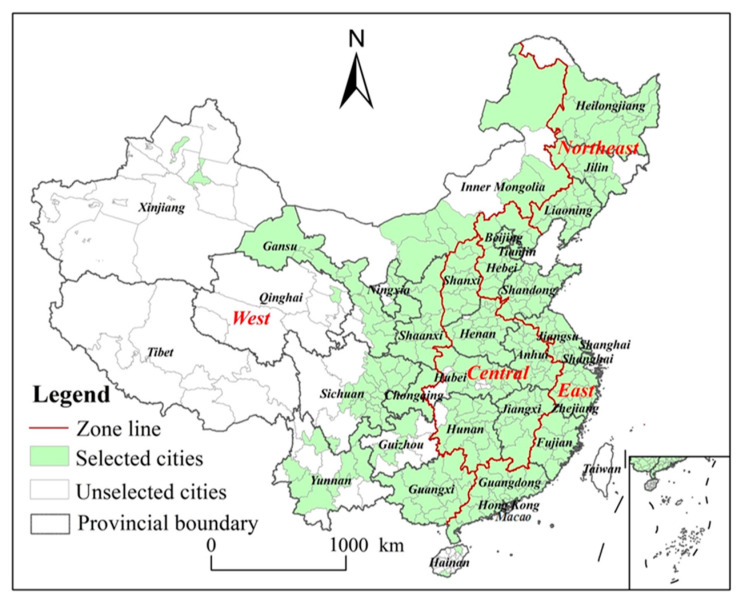
Distribution of the 283 selected cities in China.

China’s administrative regions are divided into following levels: 34 first-level (provinces), 334 s-level (prefectures), and 2851 third-level (counties). Regionally, China is divided into four main regions: eastern, central, western, and northeastern China [42]. In order to explore the change dynamics and influencing factors of CEE in prefecture-level cities in China, considering the limitation of data unavailability, 283 complete analysis sample cities were used, which account for more than 84 percent of the country’s total. The spatial distribution of the sample cities is shown in Figure 2.

## 4. Methods

In this study, the US-SBM model is firstly used to calculate the CEE of Chinese cities from 2005 to 2017. Then, the ESDA model is used to calculate the spatial static agglomeration characteristics of CEE, the Spatial Markov chains is used to measure the dynamic evolution characteristics of CEE types, and then, the entropy method is used to calculate the multidimensional urbanization development index. Finally, the GTWR model is used to calculate the spatiotemporal impact of multidimensional urbanization on CEE.

### 4.1. US-SBM Model

In the process of economic production, the input of capital, labor force, and energy not only produces industrial products, but also the undesirable output. The US-SBM model, which is proposed by Tone [43], solves the problem of relaxation improvement of input-output factors and can classify effective decision-making units (DMU). Therefore, this study uses the US-SBM model to evaluate the CEE. We assume that there are n decision-making units (DMU), and each unit consists of three factors: inputs, desirable outputs, and undesirable outputs. Each unit uses m input factors to produce c1 desirable outputs and c2 undesirable outputs. We define the matrix: $X=\left[x_{1},x_{2},\cdots ,x_{n}\right]\in R_{m\times n}$, $X=\left[x_{1},\cdots ,x_{n}\right]\in R_{m\times n}$, $Y^{g}=\left[y_{1}^{g},y_{2}^{g},\cdots ,y_{n}^{g}\right]\in R_{c1\times n}$, $Y^{g}=\left[y_{1}^{g},\cdots ,y_{n}^{g}\right]\in R_{s1\times n}$, and $Y^{b}=\left[y_{1}^{b},y_{2}^{b},\cdots ,y_{n}^{b}\right]\in R_{c2\times n}$, $Y^{b}=\left[y_{1}^{b},\cdots ,y_{n}^{b}\right]\in R_{s2\times n}$, $x\in R_{m}$, $y^{g}\in R_{c1}$, $y^{b}\in R_{c2}$, $x\in R_{m}$, $y^{g}\in R_{s1}$, $y^{b}\in R_{s2}$. Then, the production possibility set can be described as follows:

(1) $$X=\left[x_{1},\cdots ,x_{n}\right]\in R_{m\times n},\;Y^{g}=\left[y_{1}^{g},\cdots ,y_{n}^{g}\right]\in R_{s1\times n}\;\;P\backslash \left(x_{0},y_{0}^{g},y_{0}^{b}\right)=\left\{\left(\bar{x},{\bar{y}}^{g},{\bar{y}}^{b}\right)|\bar{x}\geq \overset{n}{\underset{k=1}{\sum }}\lambda_{k}x_{k},{\bar{y}}^{g}\leq \overset{n}{\underset{k=1}{\sum }}\lambda_{k}y_{k}^{g},{\bar{y}}^{b}\geq \overset{n}{\underset{k=1}{\sum }}\lambda_{k}y_{k}^{b},\lambda \geq 0\right\}.\;$$

The US-SBM model is detailed as follows:

(2) $$\rho^{*}=\min\frac{\frac{1}{m}\sum_{i=1}^{m}\frac{\bar{x_{i}}}{x_{i0}}}{\frac{1}{c_{1}+c_{2}}\left(\sum_{r=1}^{c_{1}}\frac{{\bar{y}}_{r}^{g}}{y_{r0}^{g}}+\sum_{l=1}^{c2}\frac{{\bar{y}}_{u}^{b}}{y_{u0}^{b}}\right)}\;s.t.\left\{ \begin{array}{l} \bar{x}\geq \sum_{k=1,\neq 0}^{n}\lambda_{k}x_{k},k=1,\cdots ,m\\ {\bar{y}}^{g}\leq \sum_{k=1,\neq 0}^{n}\lambda_{k}y_{k}^{g},r=1,\cdots ,c_{1}\\ {\bar{y}}^{b}\geq \sum_{k=1,\neq 0}^{n}\lambda_{k}y_{k}^{b},u=1,\cdots ,c_{2}\\ \bar{x}\geq x_{0},{\bar{y}}^{g}\leq y_{0}^{g},{\bar{y}}^{b}\geq y_{0}^{b}\\ \lambda \geq 0,\sum_{k=1,\neq 0}^{n}\lambda_{k}=1 \end{array}\right.$$

where $\rho^{*}$ is the efficiency value; x, $xy^{g}$, and $y^{b}y^{b}$ are the vectors of the input, ideal output, and undesirable output, respectively. $s^{-}s^{-},\;s^{g}s^{g}$, and $s^{b}s^{b}$ are the relaxation improvement of the input, ideal output, and undesirable output, respectively; and $\rho^{*}$ is greater than 0.

In the literature on carbon emission performance accounting, the generally considered important input factors are capital, labor, and energy [38,39,40,44,45]. Drawing lessons from existing research, the capital investment and investment in fixed assets at the end of each city are selected as the labor input, the urban energy consumption as energy input, GDP as the expected output, and carbon emissions as the expected output. The constructed CEE input-output index system of Chinese cities is shown in Table 1.

### 4.2. Global Spatial Autocorrelation Analysis

The global spatial autocorrelation model can effectively reflect the static spatial distribution of city CEE. Moran’s I of global spatial autocorrelation is used to represent the spatial correlation degree of CEE in each region. The calculation method is as follows [46]:

(3) $$Moran’sI=\frac{\sum_{i=1}^{n}\sum_{j=1}^{n}\omega_{ij}\left({\mathrm{CEE}}_{i}-\bar{\mathrm{CEE}}\right)\left({\mathrm{CEE}}_{j}-\bar{\mathrm{CEE}}\right)}{S^{2}\sum_{i=1}^{n}\sum_{j=1}^{n}\omega_{ij}}$$

where *n* is the total number of space units; CEE_i_ and CEE_j_ represent the values of space *i* and *j*, respectively; *w_ij_* represents the spatial weight; $\bar{\mathrm{CEE}}$ and CEE is the mean value and S^2^ is the sample variance. The value of *Moran’s I* is within the interval [–1, 1]. If the value is close to 1, it is a spatially positive correlation; otherwise, it is a spatially negative correlation. When the value is close to 0, it means that CEE is randomly distributed and has no spatial autocorrelation. In addition, both Z and *p* values are used for the statistical analysis. Moreover, the heat analysis tool in ArcGIS 10.2 software is used to analyze the Getis-Ord Gi* index, and the natural breakpoint method is used to explore the hot and cold spots of CEE in Chinese cities [47].

### 4.3. Spatial Markov Chains

The spatial dynamic spillover effect of CEE between cities can be analyzed through Markov chains. The CEE is divided into k types, and then the probability of the total number of cities occupied by each type and its transition probability are calculated respectively. Element m_ijt_ represents the probability that the CEE type of a city changes from year *t* to year *t* + 1. The probability matrix relation of adjacent years can be expressed as:

(4) $$R_{t+1}=M\times R_{t}$$

where *M* is the transfer matrix of K*K and *Rt* is the probability matrix of *K* types in year *t*.

Regions are not isolated from each other, and a region is often influenced by its neighboring regions. Therefore, spatial Markov chain introduces “spatial lag” to represent the neighborhood state of a region, which to some extent makes up for the deficiency that Markov chain cannot take spatial into account [48]. The specific formula is as follows:

(5) $$Lag_{a}=\sum^{}Y_{b}W_{ab}$$

where *Lag_a_* is the spatial lag value of the region a; *Y_b_* represents the attribute value of the region; and *W_ab_* represents the spatial weight matrix, that is, the spatial relationship between region a and region b.

### 4.4. Evaluating the Urbanization Level

A single index method and comprehensive index method are used to measure urbanization. The single indicator method, such as the proportion of non-agricultural population [38], can only measure the transfer of the rural population to cities. Urbanization is a complex and dynamic process, indicating the comprehensive development of economy, land, infrastructure, and social services. Urbanization can be regarded as a concentrated manifestation of human economic and social activities [10]. In recent years, researchers have tended to construct scientific comprehensive indicators, including population urbanization (PU), economic urbanization (EU), spatial urbanization (SPU), and social urbanization (SU), to measure the regional urbanization development level [49,50,51,52]. On this basis, combined with the Chinese government putting forward the basic characteristics of urbanization, the integrated urban and rural areas, the industrial interaction, intensive development, and ecological livable are considered to develop the urbanization evaluation system. Based on the availability of data, the urbanization evaluation is finally determined from the four dimensions of population, economy, space, and society, as shown in Table 2.

According to the entropy method, the urbanization of various dimensions and the comprehensive urbanization index are calculated. All indicators are standardized to make different variables comparable by using the following formulas:

(6) $$y_{+ij}=\left(x_{ij}-x_{ijmin}\right)/\left(x_{ijmax}-x_{ijmin}\right),\;$$

(7) $$y_{-ij}=\left(x_{ijmax}-x_{ij}\right)/\left(x_{ijmax}-x_{ijmin}\right),\;$$

where *y_+ij_* represents the positive indicator; *y_−ij_* represents the negative indicator; *x_ij_* represents the value of indicator *j* in city *i*; and *x_ijmax_* and *x_ijmin_* indicate the maximum and minimum value of the indicator *j*, respectively. Then, the entropy weight calculation is used to determine the importance of each indicator:

Firstly, to calculate the sample indicator weight:

(8) $$p_{ij}=x_{ij}/\sum_{i=1}^{n}x_{ij},\;$$

Secondly, to calculate the entropy of indicator *j*:

(9) $$e_{j}=-k\sum_{i=1}^{n}p_{ij}\times \ln p_{ij},\;$$

(10) k=1/lnn, 

Thirdly, to calculate the utility value of each indicator:

(11) $$d_{j}=1-e_{j},\;$$

Finally, to calculate the indicator weight:

(12) $$w_{j}=d_{j}/\sum_{j=1}^{n}d_{j},\;$$

The linear weighting method is used to calculate the urbanization development index of the city:

(13) $$U_{i}=\overset{n}{\underset{j=1}{\sum }}w_{j}\times y_{ij}$$

where *U_i_* refers to the development index of comprehensive urbanization (*CU_i_*), population urbanization (*PU_i_*), economic urbanization (*EU_i_*), spatial urbanization (*SPU_i_*), and social urbanization (*SOU_i_*).

### 4.5. Geographically and Temporally Weighted Regression (GTWR)

This study adopts the GTWR model to reveal the influencing factors of multidimensional urbanization on the CEE spatial spillover effect. The GTWR model adds the time dimension and considers the non-stationary characteristics of time and space, which makes the estimation more accurate, and it can reflect the spatio-temporal heterogeneity of different regions [53,54]. The basic formula is as follows:

(14) $$y_{i}=\beta_{0}\left(long_{i},lat_{i},t_{i}\right)+\sum_{j}^{n}\beta_{j}\left(long_{i},lat_{i},t_{i}\right)x_{ij}+\varepsilon_{i}$$

where *y_i_* is the CEE of city *i*; *x_ij_* represents the value of city *i* for the *k*-th explanatory variable of CEE, CU, PU, EU, SPU, and SOU are used as core explanatory variables. $long_{i}$ and $lat_{i}$ are the longitude and latitude coordinates of city *i*, and *t_i_* is the year *t*. $\beta_{0}\left(long_{i},lat_{i},t_{i}\right)$ is the intercept term, $\beta_{j}\left(long_{i},lat_{i},t_{i}\right)$ is the estimated coefficient of explanatory variable; $\varepsilon_{i}\overset{iid}{\sim }N\left(0,\sigma^{2}\right)$ is a random error term. The estimated value of each regression coefficient of city i is:

(15) $$\hat{\beta }\left(long_{i},lat_{i},t_{i}\right)={\left[X^{T}W\left(long_{i},lat_{i},t_{i}\right)X\right]}^{-1}X^{T}W\left(long_{i},lat_{i},t_{i}\right)Y$$

where $\hat{\beta }\left(long_{i},lat_{i},t_{i}\right)$ is the estimated value of $\beta_{j}\left(long_{i},lat_{i},t_{i}\right)$ and $W\left(long_{i},lat_{i},t_{i}\right)$ is the space-time weight matrix. *X* is the matrix formed by independent variables; *X^T^* is the transpose of the matrix; *Y* is the matrix of observed values.

In order to avoid the “long tail effect” caused by data discreteness, this study combined the distance threshold method and Gaussian function method commonly used in weight determination, and adopted the finite Gaussian function, namely bi-square spatial weight function [55]:

(16) $$W_{ik}^{ST}={\{\begin{array}{c} \left[1-{\left(\frac{d_{ik}^{ST}}{b_{i}}\right)}^{2}\right]\\ 0,\;d_{ik}^{ST}>b_{i} \end{array}}^{2},d_{ik}^{ST}\leq b_{i}$$

where *W* is the space-time weight matrix obtained by using the *b_i_*-square spatial weight function, and *d^s^* is the space-time distance between observation point *i* and *k*. In Formula (6), Bandwidth B will affect the establishment of the space-time weight matrix. Considering the density of the data observation point distribution, adaptive bandwidth is adopted in this study, and the established criterion is AICc criterion [55].

## 5. Results and Discussion

### 5.1. CEE in Chinese 283 Cities

The CEE of Chinese cities has obvious differences in space. From the average CEE of 283 prefecture-level cities, it improved from 2005 to 2017, and the average CEE is 0.4693. Shenzhen, Ordos, Maoming, Guangzhou, Suihua, and Ziyang achieved relatively high ecological benefits, exceeding 1. The eco-efficiency in some cities, including Fuxin, Guyuan, and Hegang, is relatively low, lower than 0.3.

**Figure 3:**
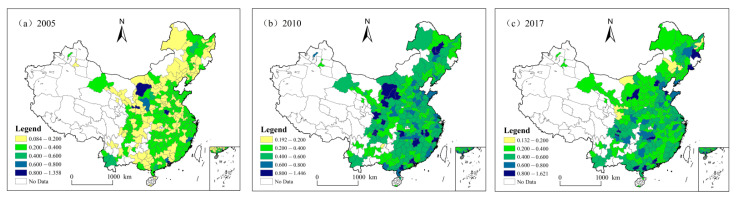
Spatial distribution pattern of carbon emission efficiency at the city level in China, in (**a**) 2005, (**b**) 2010, and (**c**) 2017.

In order to understand the evolution trend of CEE from a spatial perspective, three representative CEE data in 2005, 2010, and 2017 are selected, and the spatial patterns are divided into five categories: low, relatively-low, medium, relatively-high, and high. The spatial distribution is shown in Figure 3. The CEE efficiency in 2005 is concentrated in low and relatively-low types. The efficiency improved in 2010 and 2017, concentrated in the relatively-low and middle categories. Specifically, five cities had high CEE in 2005, mainly concentrated in Inner Mongolia, Gansu, Henan, and Guangdong. In 2010, there were 21 CEE high cities, mainly concentrated in western regions (Inner Mongolia, Gansu, Shaanxi, Shanxi, Sichuan), central regions (Hubei, Jiangxi), eastern regions (Fujian, Guangdong), and northeast regions (Heilongjiang). By 2017, the number of CEE high cities decreased to 13, mainly concentrated in the east (Beijing, Shandong, Zhejiang, Guangdong), central (Henan, Anhui, Hunan), northeast (Jilin, Heilongjiang), and west regions (Sichuan). It can be found that from 2005 to 2017, the CEE efficiency of each city constantly transformed to s high type, but the number of efficient cities increased first and then decreased, and the cities with high efficiency gradually shifted from the west to the east. Due to the capital endowment and industrial structure of eastern cities, it is easier to promote the transformation to an energy-saving industry, thus improving CEE. However, some resource-based cities in western China have a single industrial structure and high dependence on fossil energy, and their CEE gradually decreases in the process of low-carbon transformation.

### 5.2. Spatial Static Agglomeration Characteristics of CEE

Table 3 shows the changes of the global Moran’s I index during the whole exploration period. Except 2005, all the years passed the test at least at the significance level of 10%. From 2005 to 2017, the global Moran’s I index showed an upward trend of fluctuation. It indicates that the spatial agglomeration of CEE gradually increased. The energy use efficiency and technology level between neighboring cities have a certain spillover effect. Meanwhile, the exchange and cooperation between cities tend to be stable, and the change of the spatial agglomeration level is becoming smaller. These results show that CEE is not independent in space, as it presents significant spatial clustering and a spatial positive correlation phenomenon.

**Figure 4:**
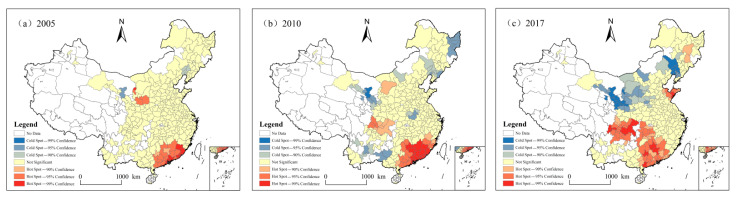
Getis-Ord Gi* maps for carbon emission efficiency at the city level in China, in (**a**) 2005, (**b**) 2010, and (**c**) 2017.

Moran’s I index can only test spatial agglomeration on the whole. The spatial visualization of the Getis-Ord Gi* statistical index is carried out by using ArcGIS10.2 to reveal the clustering distribution pattern of CEE, and also show the evolution characteristics of hot spots and cold spots, as shown in Figure 4. From 2005 to 2017, the number of hot and cold spots in CEE increased over time, indicating CEE spatial autocorrelation is enhanced in Chinese cities. Among them, with the exception of 2005, the hot spots’ influence area has been expanding and showing a transition from north to south. Specifically, the hot spots of CEE spatial agglomeration in 2005 are concentrated in the southeast coastal area and Shaanxi-Gansu-Ningxia region. To be specific, from 2005 to 2010, hot spots in southeastern coastal areas spread to surrounding areas, and hot spots in western areas evolved to Inner Mongolia and Sichuan-Chongqing regions. Cold spots are scattered in northeast China; Henan and Anhui in central China; and Gansu, Guangxi, and Yunnan in western China. From 2010 to 2017, hot spots appeared in Shandong, while hot spots in Chengdu-Chongqing and the southeastern coastal areas extended to Hunan. The cold spots are concentrated in northern China (Liaoning, Inner Mongolia, Shanxi, Shaanxi, Gansu, Ningxia). The overall level of economic development in these regions is relatively low and in the transitional development stage, and the demand for energy relatively low.

### 5.3. Spatial Dynamic Agglomeration Characteristics of CEE

Although spatial autocorrelation analysis reveals the global or local spatial correlation clustering characteristics of CCE, it does not consider the dynamics of spatial spillover effects. Comparing the results of the Markov transfer matrix and spatial Markov transfer matrix will help to reveal the dynamic spatial spillover effect of CEE. The CCE from 2005 to 2017 is divided into four types: low level, medium-low level, medium-high level, and high level, by using the quartiles method, which are represented by k = 1, 2, 3, 4, respectively. For comparative analysis, the development process of CCE is divided into two stages: 2005–2010 and 2011–2017 (Table 4).

In Table 4, the elements on the diagonal line represent the probability of maintaining the original state type of the urban carbon emission type, while the elements on the non-diagonal line represent the probability of state transition. The lowest value of the diagonal in the two time periods is 0.3601, and the highest value of the non-diagonal is 0.2694. The probability value of the diagonal is greater than the probability value of the non-diagonal, indicating that the type transfer of CEE is stable and the probability of maintaining the original state is high. In addition, there is a phenomenon of “club convergence” in the CEE of Chinese cities. For example, the probability of low and high CEE from 2011 to 2017 maintaining their original status in the next stage is the highest, which is 0.7722 and 0.6977, respectively. In general, the probability of CCE type transfer increased by 1.1198 from 2005 to 2010 but decreased by 0.5948. From 2011 to 2017, the probability of transfer increase is 0.7915, and the probability of transfer decrease is 0.7584. The results of the two-stage transfer show that the development of the CCE type is on the rise, but the negative spillover effect of CCE type transfer in the second stage is significantly higher than that in the first stage.

The spatial Markov transfer matrix of the CEE types is shown in Table 5. By comparing Table 1 and Table 2, it is found that CEE types in the neighborhood have a significant impact on the development of CEE types in the region. From 2005 to 2010, when the city is adjacent to the region with low CEE, the probability of downward transfer of the city’s CEE type decreases, for example, P21(0.1042) > P21|1(0.0548), P32(0.2377) > P32|1(0.0435). When the city is adjacent to the region with high CEE, the probability of upward transfer of the CEE type of the city will decrease, for example, P12(0.2694) > P12|4(0.2353), P23(0.2083) > P23|4(0.1818). In contrast with 2005–2010, 2011–2017 shows a “club convergence” effect. When the city is adjacent to the region with low CEE, the probability of downward transfer of the CEE type increases, as shown P21(0.2009) < P21|1(0.2653), P32(0.2092) < P32|1(0.2535). For the neighbors of the region with high carbon emissions performance, the city’s carbon emissions performance type upward shift probability will increase, such as P12(0.2152) < P12|4(0.2424), P23(0.2757) < P23|4(0.3148).

Through the comparative analysis of the ordinary Markov and spatial Markov probability transfer matrix, it is found that the CEE of neighboring cities has a significant impact on the development of the city itself, and the impacts are different in different time periods. In particular, the CEE of Chinese cities from 2011 to 2017 is affected by the spillover effect of neighborhood types, forming a phenomenon of “club convergence”, and cities clustered in geographical space tend to shift in the same direction.

### 5.4. Analysis on the Impact of Multidimensional Urbanization on CEE in China

#### 5.4.1. Results Tests

The CEE has obvious spatial and temporal heterogeneity, thus it is necessary to consider this heterogeneity when exploring its influencing factors. Therefore, the GTWR model is introduced to explore the spatio-temporal evolution of multidimensional urbanization’s impact on CEE. The study period is divided into two periods, 2005–2010 and 2011–2017, and the GTWR results are compared with the OLS and GWR models to verify the applicability and accuracy of the GTWR model. *R^2^* and adjusted *R^2^* reflect the fitting degree of the model, and the sum of squares of residual errors (RSS) reflects the size of the model accuracy. AICc information can be used as another important criterion to evaluate the fit quality of the model: the smaller the value is, the higher the model accuracy is [56]. Table 6 shows that the fitting degree of the GTWR model in two time periods is 0.7398 and 0.6078, respectively, which is greatly improved compared with OLS and GWR. AICc values are reduced to 2769.9600 and 4016.9100, respectively, indicating significant differences between the models. RSS decreased from 1023.7115 and 1372.6223 of OLS to 441.7600 and 776.8870 of GTWR, indicating that the accuracy of the GTWR model is relatively high. We took the mean values of the regression coefficients of the two stages respectively, and it can be seen from the quanta table of the regression coefficients (Table 7) that the parameter estimates of the respective variables differ greatly, with positive and negative values, and the intensity changes obviously, indicating that the influence intensity of CEE in Chinese cities is obviously non-stationary in both time and space.

#### 5.4.2. Spatial and Temporal Distribution of Urbanization’s Impact on CEE

**Figure 5:**
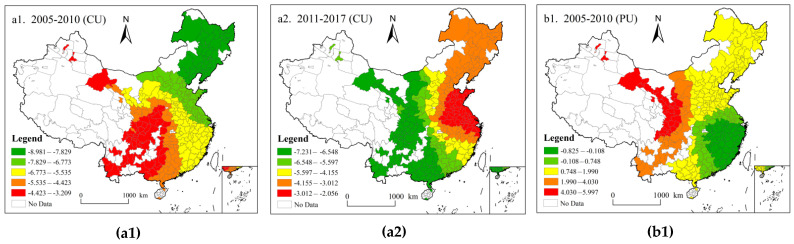

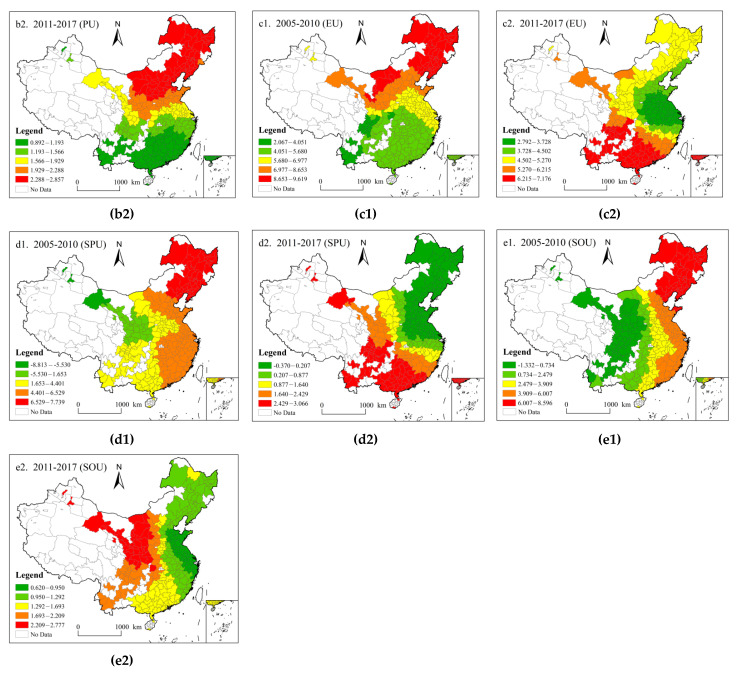
Spatial and temporal distribution of the influence of multidimensional urbanization on CEE from 2005 to 2017.

According to the regression results of the GTWR model and with reference to the work of Zhang and Chen [41,56], the natural fracture Jenks method of ArcGIS is used to divide the data with the highest similarity of the average regression coefficients of various driving factors during the sample period into the same level, and generate the spatial distribution map of driving factor coefficients (Figure 5).

1. Spatio-temporal heterogeneity of CU’s influence on CEE.

The regression coefficients of CU to CEE are shown in Figure 5(a1,a2). During 2005–2010 and 2011–2017, the influence of CU on CEE showed obvious spatial dependence, and the regression coefficients are all negative. In 2005–2010, the regression coefficient is a gradient distribution pattern: along the northeast-southwest direction decreases, and the coefficient of the largest in the northeast region distribution. Due to the rapid development of urban industrialization in northeast China and the rapid development of heavy industry dependent on energy consumption, the negative impact on carbon emission efficiency is great.

From 2011 to 2017, the CU regression coefficient of all cities decreased significantly, indicating that the low-carbon city and new-type urbanization policy implemented by the Chinese government had achieved initial results. With the implementation of the policy, the urbanization development is more intensive, and the negative impact on CEE gradually weakened. This result is similar to that of Li et al. [57], who pointed out that there is a U-shaped relationship between urbanization and carbon emission efficiency. The regression coefficient decreases along the west-central-east direction, and the negative impact of urban development on CEE in the western region is significantly higher than that in the eastern region. In the process of rapid urbanization, a large amount of infrastructure construction and energy use has led to a large number of carbon emissions, which have an increasingly negative impact on the CEE, in the western region with the implementation of the western development policy.

Due to the complex multidimensional characteristics of urbanization, it is difficult to find the specific influence path and mechanism of the single study on the impact of urbanization on CEE. It is necessary to conduct a study from a multi-dimensional perspective, so as to clarify the specific factors driving or inhibiting CEE in the process of urbanization. Therefore, this study further explores the influence of various dimensions of urbanization on CEE and its spatial-temporal variation characteristics.

2. Spatio-temporal heterogeneity of PU’s influence on CEE.

The regression coefficients of PU on CEE are shown in Figure 5(b1,b2). From 2005 to 2010, the regression coefficients of PU ranged from −0.825 to 5.997, showing a decreasing spatial distribution from northwest to southeast. Among them, the coefficients of PU in the southeastern coastal regions (the Yangtze River Delta, the western bank of the Straits, and the Pearl River Delta urban agglomerations) and some urban agglomerations in the middle reaches of the Yangtze River are negative, indicating that the population development in this region is in the stage of scale agglomeration. People migrate from the west to the southeast coastal areas, resulting in the accelerated growth of the population density. The pressure of the population bearing indirectly acts on the urban internal ecosystem and over-absorbs urban internal resources and services, resulting in the “congestion effect” [58], which has a negative impact on the urban CEE and reduces the CEE.

However, the outflow of human resources in labor-exporting provinces, such as western and central regions, is prominent, which leads to the decline of the urban population density in a certain period, and PU plays a role in promoting CEE. The comparison between 2011–2017 and 2005–2010 shows that the impact of PU on CEE presents a spatio-temporal change characteristic from the east-west to the south-north distribution. Moreover, PU in China has a significant promoting effect on CEE, indicating that the accumulation of human capital at this time brings technological progress and the use of clean technology effectively promotes the improvement of carbon emission efficiency [51,56].

3. Spatio-temporal heterogeneity of EU’s influence on CEE.

The influence coefficients of EU on CEE are shown in Figure 5(c1,c2). In both periods, the elasticity coefficient of economic urbanization has a significant positive effect on CEE. Specifically, the promotion effect of economic urbanization on CEE in northern China shows an inverted U-shaped development trend of first increasing and then decreasing, which is similar to the research conclusion of Sun et al. [38]. From 2005 to 2010, the economic benefit achieved by the improvement of productivity is greater than the carbon emission effect caused by the expansion of economic production [59]. Therefore, economic improvement has a positive impact on CEE to a certain extent.

Based on realistic evaluation, compared with the north, the economy of southern cities is dominated by tertiary industries, such as technology, finance, and service, and the industrial layout of most regions is green, sustainable, and environmentally friendly, with high economic benefits. For example, the Pearl River Delta urban agglomeration, with its superior capital endowment and infrastructure advantages, can make “big strides” and make rapid and steady progress in transformation and adjustment. The steady improvement of clean industry also promotes the continuous improvement of carbon emission efficiency. Therefore, northern cities can explore their own industrial transformation.

4. Spatio-temporal heterogeneity of SPU’s influence on CEE.

The influence coefficients of SPU on CEE are shown in Figure 5(d1,d2). From 2005 to 2010, the largest regression coefficient is distributed in Shaanxi, Gansu, Ningxia, and Karamay and other resource-based cities in Xinjiang, and the regression coefficient is negative. Resource-based cities rely on natural resources as the leading industry and have a single economic structure. For example, the proportion of the secondary industry in Karamay’s GDP even reaches 89.34%, forming a development mode of “mining first, then city”. Industrial development drives the rapid urban construction. Demand stimulates the construction of infrastructure, such as housing and public services [60], which inevitably increases resource consumption and thus promotes carbon dioxide emissions. In addition, urban expansion occupies a large amount of land, and the reduction of forest land and grassland reduces the carbon sink to a certain extent [61]. The regression coefficient of SPU in eastern and central China is positive, indicating that the urban expansion in eastern and central China is resource-conserving expansion, paying attention to the construction of ecological environment infrastructure, and promoting the improvement of CEE.

From 2011 to 2017, the regression coefficient is lower than the previous period, indicating that the promoting effect of SPU on CEE is gradually weakened. Bohai sea and part of the central city of the regression coefficient is negative, the region’s urban space is for the expansion of the low level, with industrial structure adjustment, the problem of unemployment of come off sentry duty, resulting in the loss of population being serious, loss of population and urban expansion imbalances, making the urban expansion in high input, high consumption, and low output cycle, cause the SPU’s influence on CEE for a negative marginal effect.

5. Spatio-temporal heterogeneity of SOU’s influence on CEE.

The influence coefficients of SOU on CEE are shown in Figure 5(e1,e2). From 2005 to 2010, SOU in the western region has an inhibiting effect on CEE, while in other regions, it has a promoting effect on CEE. On the one hand, many developed regions in the east, such as Shanghai and Guangdong, import most consumer goods, such as electricity, from the less developed western regions. Due to the weak production technology in the less developed regions, this phenomenon brings more carbon dioxide emissions to the production areas and reduces the CEE. On the other hand, due to the eastern region with the higher education level, it promotes residents to develop low-carbon consumer consciousness, green travel, etc. Meanwhile, a higher level of education of city innovation ability promotes the development of a green economy and efficiency, eases worsening pollution, and improves the living environment.

From 2011 to 2017, the spatial distribution characteristics of SOU’s influence on CEE are contrary to the previous period, showing a change characteristic of western China being larger than central China and larger than eastern China, and SOU’s promoting effect on CEE is found throughout the country. This shows that after the implementation of the low-carbon city policy in 2010, more and more people began to adopt low-carbon lifestyle, and urban industrialization slowly began to transform into a knowledge-based economy and clean industrial structure, which slowed down the growth rate of carbon emissions.

## 6. Conclusions and Policy Implications

This study used the US-SBM model to scientifically measure CEE, and integrated the ESDA and spatial Markov probability transfer matrix to discuss spatial agglomeration and type evolution characteristics of CEE from static and dynamic perspectives, which was supported by the panel data of 283 cities in China from 2005 to 2017. To understand the driving factors of China’s CEE spatio-temporal pattern evolution, this study also used the GTWR model to investigate the heterogeneity of the impact of multidimensional urbanization on CEE. Some of the main findings are as follows:

(1) During the sample period, CEE of Chinese cities showed a trend of gradual improvement, and the gap is significantly narrowed, but the average CEE is 0.4693. More efforts should be made to achieve the optimal efficiency; CEE high-level cities are mainly distributed in areas with relatively developed economy or resources, such as Shenzhen and Ordos. CEE low-level cities are mainly distributed in northeast China, such as Fuxin and Hegang. The number of CEE high cities increases first and then decreases, and gradually transfers from the west to the east and from the north to the south.

(2) CEE has a significant positive correlation in spatial agglomeration. The number of CEE’s “hot spot” agglomeration area and “cold spot” agglomeration area both increased over time, and the spatial autocorrelation is enhanced. The “hot spot” agglomeration area shifted from west to east and from north to south, mainly concentrated in Shandong Peninsula, Pearl River Delta urban agglomeration, and Chengdu-Chongqing urban agglomeration. The “cold spot” agglomeration area shifted from the south to the north, mainly concentrated in Inner Mongolia, Shaanxi, Gansu, and Ningxia.

(3) The spatial Markov probability transfer matrix of the CEE type shows that the CEE type is generally on the rise, with obvious spatial spillover, and different time periods have different effects. Especially, from 2011 to 2017, CEE of Chinese cities is affected by the neighborhood type spillover effect and formed the phenomenon of “club convergence”, that is, if the city is adjacent to high-level cities, the probability of CEE’s upward transition will increase, and if adjacent to low-level cities, it may inhibit the probability of CEE’s transition to a high-level stage.

(4) The impact of urbanization on CEE has spatial heterogeneity. Comprehensive urbanization has a negative impact on CEE. Economic urbanization has a significant impact on CEE, and the overall impact of economic urbanization in northern cities on CEE shows an inverted “U” shape. The impact of population urbanization on CEE shows a positive promoting effect after 2010. The impact of spatial urbanization on CEE shows obvious spatial heterogeneity, and the cities with a negative impact are distributed in the resource-based cities in northwest China and the Bohai Rim. Social urbanization has a positive promoting effect on CEE after 2010.

The above research conclusions of this study have important policy significance for promoting CEE upgrading and narrowing regional differences in China:

(1) Local governments should pay attention to the balanced development of various factors within the population, and promote the transformation of population development to optimize the structure and improve the quality of the population. (2) Cities dominated by secondary industries need to gradually improve production technology, increase production efficiency, and reduce carbon emissions throughout the process. Meanwhile, policy support for green industries should be increased to promote the formation of low-carbon industrial, such as new energy and leisure tourism. (3) Urban managers should promote the space intensive and green development of urban construction. According to the development idea that urban expansion is compatible with social and economic development to delimit the forbidden area, restricted area, and allowed construction area. (4) The lockdown caused by the COVID-19 pandemic has dramatically reduced carbon emissions around the world, prompting further thinking about the sustainability of development. Some governments have introduced a series of green development policies, such as promoting the development of new-energy vehicles, hydropower, wind, and photovoltaic power. Meanwhile, as proposed by Camerin et al. [62], exploring the urban planning and development mode of “15-min city” and “super block” to promote the construction of low-carbon demonstration and pilot projects in communities and families, popularizing the concept of low-carbon consumption, and creating a good low-carbon social atmosphere, so as to build a healthier, safer, low-carbon, and socio-economic ecological balance city.

In this study, 283 prefecture-level cities in China were taken as the research objects, and the GTWR model was used to analyze the impact of multi-dimensional urbanization on CEE, providing a decision-making basis for low-carbon city development. However, there are some limitations to this study. As for the research data, we only analyzed the CEE evolution dynamics from 2005 to 2017 based on accessibility, and the policy enlightenment from it may lag behind the actual development. Some important studies, such as Du et al., also adopted the data of this period, showing the limitations of the data [63]. Therefore, we also suggest that the establishment of timely and effective data systems should be accelerated at the city level, especially in the vast majority of cities with a rapid urbanization process except for developed cities, such as Beijing and Shenzhen. Our ongoing research is also looking for appropriate processing methods to address this limitation, such as fitting nighttime light data. On the other hand, urban development involves many aspects. As it is difficult to quantify urban planning, morphology, government restrictions, and other factors, they have to be abandoned in modeling. The COVID-19 epidemic also highlights the healthy development in the process of urbanization. Future studies will further improve the indicator system and promote sustainable urban development.

## Figures and Tables

**Table 1:** Urban CEE evaluation index system.

Variables	Indicators	Description
Input variables	Capital invested	Gross investment in fixed assets
	Labor force	Number of employees at the end of the year
	Energy consumption	Urban Standard coal consumption
Desirable output	GDP	Gross regional domestic product
Undesirable output	Carbon emissions	Amount of carbon emissions

**Table 2:** Evaluation index system of a comprehensive level of urbanization.

System	Subsystem	Specific Indicators	Unit	W1	W2
Comprehensive Urbanization (CU)	Population Urbanization (PU)	Proportion of urban population	%	0.287	0.045
		Population density	10,000 people/km^2^	0.590	0.096
		Proportion of persons employed by secondary and tertiary industries	%	0.123	0.032
	Economic Urbanization (EU)	Per capita GDP in urban areas	Yuan	0.426	0.049
		Ratio of urban and rural per capita disposable income (-)	%	0.235	0.084
		Proportion of the added value of the second industry to GDP (-)	%	0.225	0.056
		proportion of the added value of the tertiary industry to GDP	%	0.114	0.024
	Spatial Urbanization (SPU)	Proportion of urban area to total area	%	0.130	0.032
		Urban built-up area per capita	km^2^/10,000 people	0.319	0.129
		Road area per capital	km^2^/10,000 people	0.246	0.094
		Urban unit area Net Primary Productivity	gC/(m^2^·a)	0.304	0.078
	Social Urbanization (SOU)	Per capita total retail sales of consumer goods in urban areas	Yuan/person	0.304	0.043
		Number of hospital beds per 10,000 persons	One/10,000 people	0.246	0.078
		Number of college students per 10,000 persons	One/10,000 people	0.319	0.114
		Number of buses per 10,000 persons	One/10,000 people	0.130	0.047

Note: (-) indicates that this indicator is a negative indicator, W1 is the weight of urbanization indicators in all dimensions, and W2 is the weight of comprehensive urbanization indicators.

**Table 3:** Moran’s I for CEE at the city level in China from 2005–2017.

Year	2005	2006	2007	2008	2009	2010	2011	2012	2013	2014	2015	2016	2017
Moran’s I	0.0046	0.0618	0.0498	0.0610	0.0422	0.0570	0.0277	0.0615	0.0268	0.0596	0.0524	0.1107	0.0998
Z-value	0.4454	3.3510	2.7266	3.1469	2.1777	2.9714	1.5660	3.2965	1.4433	3.2353	2.7697	5.7000	5.1590
*p*-value	0.287	0.002	0.005	0.009	0.026	0.007	0.069	0.005	0.076	0.004	0.009	0.001	0.002

**Table 4:** Markov transfer matrix from 2005 to 2017.

	*t\t +* 1	*n*	1	2	3	4
2005–2010	1	386	0.3601	0.2694	0.2124	0.1580
	2	336	0.1042	0.6101	0.2083	0.0774
	3	345	0.0058	0.2377	0.5623	0.1942
	4	348	0.0000	0.0316	0.2155	0.7529
2011–2017	1	395	0.7722	0.2152	0.0127	0.0000
	2	428	0.2009	0.4883	0.2757	0.0350
	3	435	0.0460	0.2092	0.4920	0.2529
	4	440	0.0182	0.0705	0.2136	0.6977

**Table 5:** Spatial Markov probability transition matrix of the CEE type in Chinese cities from 2005 to 2017.

Neighbor Type	*t\t +* 1	2005–2010					2011–2017				
		*n*	1	2	3	4	*n*	1	2	3	4
1	1	274	0.2336	0.2701	0.2883	0.2080	177	0.8192	0.1695	0.0113	0.0000
	2	73	0.0548	0.4795	0.2055	0.2603	98	0.2653	0.5000	0.1837	0.0510
	3	23	0.0435	0.0435	0.6522	0.2609	71	0.0423	0.2535	0.5493	0.1549
	4	19	0.0000	0.1579	0.2105	0.6316	37	0.0000	0.0270	0.2432	0.7297
2	1	52	0.6346	0.2692	0.0385	0.0577	91	0.7363	0.2527	0.0110	0.0000
	2	112	0.1161	0.6607	0.2054	0.0179	123	0.1545	0.5122	0.2927	0.0407
	3	104	0.0096	0.3173	0.4712	0.2019	102	0.0490	0.1863	0.5098	0.2549
	4	66	0.0000	0.0606	0.1970	0.7424	94	0.0106	0.0638	0.1809	0.7447
3	1	26	0.6154	0.3077	0.0385	0.0385	61	0.7213	0.2623	0.0164	0.0000
	2	85	0.1294	0.6118	0.2353	0.0235	99	0.1919	0.4747	0.3030	0.0303
	3	118	0.0000	0.2288	0.6017	0.1695	140	0.0429	0.1500	0.5071	0.3000
	4	120	0.0000	0.0167	0.2500	0.7333	144	0.0139	0.0625	0.2569	0.6667
4	1	34	0.7647	0.2353	0.0000	0.0000	66	0.7424	0.2424	0.0152	0.0000
	2	66	0.1061	0.6667	0.1818	0.0455	108	0.2037	0.4630	0.3148	0.0185
	3	100	0.0000	0.2100	0.5900	0.2000	122	0.0492	0.2705	0.4262	0.2541
	4	143	0.0000	0.0140	0.1958	0.7902	165	0.0303	0.0909	0.1879	0.6909

**Table 6:** Comparison of model test results.

Model	2005–2010				2011–2017			
	R^2^	Adjusted R^2^	RSS	AICc	R^2^	Adjusted R^2^	RSS	AICc
OLS	0.3967	0.3950	1023.7115	3971.4995	0.3068	0.3050	1372.6223	4907.0476
GWR	0.6690	0.6680	562.0600	3035.4700	0.4279	0.4264	1133.4200	4622.0500
GTWR	0.7398	0.7391	441.7600	2769.9600	0.6078	0.6068	776.8870	4016.9100

**Table 7:** Test results of the GTWR model.

Quantile	2005–2010					2011–2017				
	Minimum	Lower Quartile	Median	Upper Quartile	Maximum	Minimum	Lower Quartile	Median	Upper Quartile	Maximum
Intercept	−1.269	−0.905	−0.666	−0.497	−0.354	−0.959	−0.814	−0.685	−0.616	−0.577
CU	−8.981	−7.049	−5.729	−4.689	−3.209	−7.231	−6.675	−4.602	−3.232	−2.056
PU	−0.825	0.142	1.104	1.600	5.997	0.892	1.145	1.704	2.269	2.857
EU	2.067	5.124	5.970	7.659	9.619	2.792	3.835	4.713	5.947	7.176
SPU	−8.813	−5.061	2.773	5.461	7.739	−0.370	−0.056	1.019	2.451	3.066
SOU	−1.332	1.037	3.561	4.496	8.596	0.620	1.084	1.375	1.864	2.777

## Data Availability

The basic data used in the research can be found on the website of National Bureau of Statistics, China Statistical Yearbook, CEADs and other databases.
